# Temporal Precedence of Physical Literacy and Basic Psychological Needs Satisfaction: A Cross-Lagged Longitudinal Analysis of University Students

**DOI:** 10.3390/ijerph17124615

**Published:** 2020-06-26

**Authors:** Fong-Jia Wang, Chih-Fu Cheng, Mei-Yen Chen, Kim-Wai Raymond Sum

**Affiliations:** 1 Department of Physical Education, National Taiwan Normal University, 162, Section 1, Heping E. Rd., Taipei City 106, Taiwan; arno1991324@gmail.com; 2 Graduate Institute of Sport, Leisure and Hospitality Management, National Taiwan Normal, 162, Section 1, Heping E. Rd., Taipei City 106, Taiwan; 3 Department of Sports Science and Physical Education, Chinese University of Hong Kong, G09, Kwok Sports Building, Shatin, N.T., Hong Kong, China; kwsum@cuhk.edu.hk

**Keywords:** physical literacy, cross-lagged panel design, self-determination theory

## Abstract

Purpose: Building on self-determination theory and extending research in the physical education context in terms of basic psychological needs satisfaction and physical literacy, this study examines the bidirectional effects of basic psychological needs satisfaction and physical literacy over time in a university physical literacy context. Method: Using a two-wave design, data were collected twice with an 18-week time lag from a sample of 549 university students. Utilizing full cross-lagged analyses, we examined the bidirectional effects between basic psychological needs satisfaction and physical literacy. Results: The results provide support for a positive relationship between physical literacy (Time 1) and basic psychological needs satisfaction (Time 2), but we cannot reject the possibility of a bidirectional relation, supporting our expectations. Conclusion: Overall, the study highlights the importance of a reciprocal relationship between physical literacy and basic psychological needs satisfaction.

## 1. Introduction

Basic psychological needs satisfaction (BPNS), stemming from self-determination theory (SDT), posits that humans have three basic psychological needs, namely, the innate needs for autonomy, competence and relatedness, which are essential for positive human growth and optimal functioning [1,2]. This is a key point in our proposed link between physical literacy (PL) and BPNS, in that personal learning experiences are viewed as actualizing capability behaviors and serve as a foundation for autonomy and the sense of self [2,3]. The need for relatedness refers to the need to feel significant, connected to, and cared for by important others rather than feeling isolated or disconnected from others. The need for competence refers to the need to experience efficacy, mastery, and skillfulness rather than incompetence [4,5]. In the context of physical education (PE), BPNS has been found to contribute to various individual outcomes, such as lower ill-being and higher well-being [2,6], higher adaptive cognitive response [4,5], higher behavioral intention [7], as well as better behavioral persistence [8]. Because of the beneficial role in promoting quality of life and health through PE, researchers have focused on identifying factors that cultivate students’ BPNS.

Therefore, the BPNS theory [2] explains that in the PE learning process, students have better athletic performance through autonomous behavior, which also provides them with psychological support factors for physical activity while satisfying their individual psychological needs. Furthermore, students’ learning through PE courses will generate different BPNS states, thereby affecting their ability to continue with PL [9]. Consequently, we propose that BPNS will contribute to the development of PL. First, as with the concept of BPNS, individuals are born with the basic psychological needs of autonomy, feelings of competence and relationship [6], and in the special domain of PE, the satisfaction of these psychological needs contributes to the growth of the individual’s physical activity ability [3]. Among them, physical activity ability is the core element of PL [3,10]. Therefore, in the special domain of PE, the students’ PL may be affected by their interaction with the external environment according to their learning experience at different times.

Second, BPNS is an important factor that influences physical activity in PE domains, and is regarded as an important concept in testing students’ sports learning experience. There are many factors influencing a student’s PE learning process, and past studies have confirmed that behaviors through different sports environments or physical activities will promote BPNS, enhancing students’ self-efficacy, and they will demonstrate better athletic abilities as a result. In the PE learning process, individuals improve their intrinsic motivation and confidence through their own experiences of participating in sports and through their physical activity abilities, further promoting their individual PL [9]. It is worth noting that in the PE learning domain, students will generate autonomous motivations, improve the psychological support factors for practicing physical activity, and thus enhance their PL [4,11]. Past studies in PE have recognized several antecedents that influence students’ BPNS, such as autonomy support [12], social environmental contexts [2], mastery of the climate (e.g., perceive in the self the innate needs for autonomy, competence and relatedness) [13,14], and interpersonal involvement [15].

Although these studies have advanced our understanding of both theory and practice from a contextual perspective, several critical issues are rarely explored. For example, a number of studies on SDT have suggested that personal disposition would make a significant contribution to the personal BPNS quality (e.g., an important potential motivational process, basic psychological needs fulfillment that underlines the relationship between PL and BPNS) [16,17,18,19,20]; however, what seems to have been neglected is the possibility that context-specific personal factors might influence the BPNS in PE (e.g., one’s physical capabilities increase one’s learning experiences). If so, to cultivate and equip them with such abilities would be propitious to achieving positive learning experiences for students in PE classes.

In the current study, we draw upon SDT to consider whether having context-specific abilities is associated with BPNS. In particular, we suggest that PL, a disposition including the motivation, confidence, physical competence, knowledge and understanding to maintain physical activity throughout the course of one’s life [3,21,22], will play a critical role in shaping individual positive learning experiences (e.g., BPNS). In particular, individuals with higher levels of PL reflect a greater sense of self-competence [23], good social interaction [11], and better personal capability for autonomous application to physical activity [22]. As such, we expect individuals high in PL to positively shape the development of BPNS within the PE context. Moreover, because PL has been advocated by and integrated into several educational and sport policies in recent years [24], physical educators and practitioners value and enhance good learning experiences as an educational standard for PE courses to promote students’ PL [25]. Investigating how individuals’ PL is related to their BPNS may be critical for providing insights into the development of PE practice for both teachers and learners. Therefore, the BPNS theory [2] explains that in the PE learning process, students have better athletic performances through autonomous behavior, which also provides them with psychological support factors for physical activity while satisfying their individual psychological needs. The application of PL not only involves the understanding of sports and continuous participation in physical activities, but also involves the development of athletic abilities and the promotion of a healthy psyche [10]. BPNS is therefore an important factor that influences physical activity in PE domains, and is regarded as an important concept in testing students’ sports learning experience.

We further propose that students’ positive learning experiences are an important source of the development of PL. We specifically argue that a higher level of BPNS will be positively associated with an increase in PL. PL is developed from a lifelong holistic learning process in physical activity and is acquired and applied in physical activity contexts [26,27]. Moreover, PL has far-reaching applications as it is not only about understanding and engaging in physical activity, but is also about personal learning experiences and actualizing capabilities [28]. These arguments imply a contextual effect that students’ BPNS in PE may exert on PL over time. Accordingly, investigating the bidirectional process between PL and BPNS may help to explain whether BPNS contributes to the development of PL, or whether they develop mutually. The benefits of these need satisfactions have been documented in research across nations, cultures, and many life domains, including education, work, and sport [3,29].

Meanwhile, PL has been vaunted as a key disposition for students of all abilities to establish lifelong adherence to physical activity [11,22,27]. For example, it strengthens their ability to do physical activities, and their knowledge and understanding of sports, and promotes students’ application of individual athletic abilities, their understanding of the concept of sports, and their mental state while facing difficulties [11]. PL can also be an important factor in promoting students’ good learning experience in the process of PE learning [12]. In the PE domain, the BPNS generated by the student will affect the individual’s athletic abilities alongside increases in PL. However, based on the SDT perspective, PL is the individual’s ability to participate in sports in a PE-specific domain, and may also affect the student’s BPNS state in the PE domain. Therefore, it is important to clarify the direction of the relationship between BPNS and PL in a PE domain. For the foregoing reasons, we posited that PL and BPNS could have a significant influence on each other.

The purpose of this study was to examine the bidirectional effects of BPNS and PL over time in a university PE context. We consider that this study is relevant because we intend to show the causal ordering of the BPNS considered separately as well as the PL indicators. Furthermore, there are still methodological pitfalls and deficiencies in longitudinal research, which make it very difficult to detect causal as well as reciprocal relationships [30]. Thus, we cannot demonstrate causality. We can only make causal relationships plausible by ruling out alternative explanations [31]. However, we accomplished this goal by using a cross-lagged panel design—a design that has been frequently used to examine relationships between variables for which a reciprocal relationship is hypothesized. In this respect, several recommendations can be made, such as performing a full panel design with an adequately planned time lag, taking stabilities of variables into account, and using covariance structure modelling [31,32,33].

Consistent with the literature reviewed above, we have formulated three specific hypotheses:

First, PL promotes continuous physical activity among students [9,12,27]; PL is an important continuation of physical activity, and the exploration of each attribute in terms of PL’s impact on students’ individual BPNS would further consolidate the concept of PL. Therefore, our hypothesis H1 is as follows: PL at Time 1 has a positive association with BPNS at Time 2, controlling for the BPNS at Time 1.

Second, BPNS is an important factor that influences physical activity in PE domains, and individuals improve their intrinsic motivation and confidence through their own experiences of participating in sports, which further promote their individual physical activity ability [4,6,12]. In the PE learning process, students will generate autonomous motivations, improve the psychological support factors for practicing physical activity, and they will demonstrate better PL [9,11]. Therefore, our hypothesis H2 is as follows: BPNS at Time 1 has a positive association with PL at Time 2, controlling for the PL at Time 1.

Third, based on the SDT perspective, PL is the individual’s ability to perform in a PE-specific domain, and may also affect the student’s BPNS state in the PE domain. Therefore, it is important to clarify the direction of the relationship between BPNS and PL in a PE domain. For the foregoing reasons, it was expected that in the PE domain, the reciprocal relationship between PL and BPNS could have a significant influence on each other. Therefore, our hypothesis H3 is as follows: there is a reciprocal relationship between PL and BPNS.

## 2. Method

### 2.1. Participants and Procedure

In Taiwan, PE class is mandatory for all first-year university students, which means that all university students are required to take part in 120-minutes of compulsory PE classes on a weekly basis. A total of 594 university students in Taiwan (280 male, 314 female) with a mean age of 18.51 (SD = 0.97) were recruited as participants in this study. Regarding the sports expertise represented by the participants, the majority were currently engaged in a interscholastic sports team (96.3%), and regarding the weekly hours of sports, the largest proportion of student participated in sports for 1 h (103 people, 17.3%), followed by 2 h (100 people, 16.8%), under 1 h (75 people, 12.6%), 3 h (69 people, 11.6%), and 4 h (46 people, 7.7%). In all, 80.8% of the participants declared their weekly hours of self-reported physical activity to be over 2 h. To test our hypotheses, a two-wave survey with repeated measurements was employed to collect data. Specifically, the variables were measured at intervals of 18 weeks in order to confirm the reciprocal relationship among the variables. First, the letter of invitation was sent to PE teachers in each school. PE teacher consent was obtained before the two waves of questionnaires were distributed. Second, all participants were asked to provide informed consent with emphasis on the voluntary nature of the survey before participating in the study. The questionnaires were distributed to the participants before PE classes. The average range of questionnaire completion time was 10–15 minutes. Participants’ confidentiality and anonymity were assured.

### 2.2. Measurements

Perceived physical literacy instrument for adolescents (PPLI-A). Individuals’ perceived physical literacy was assessed using the PPLI-A. PPLI-A is a 9-item instrument used to measure both student and teacher participants’ PL. The three sub-scales are knowledge and understanding, sense of self and self-confidence, and self-expression and communication with others [34]. The version of the PPLI-A consisting of nine items used in this study was constructed based upon the PPLI-A developed by Sum et al. [11], and items were scored on a 5-point Likert scale (1 = strongly disagree to 5 = strongly agree). The scale consists of three subscales: “sense of self and self-confidence” (3 items), “self-expression and communication with others” (3 items), and “knowledge and understanding” (3 items). In Sum et al.’s [11] study, the confirmatory factor analysis (CFA) showed that the construct demonstrated a good fit to the model. In this study, the internal consistency reliability was 0.89. 

The Basic Psychological Needs Satisfaction (BPNS). Individuals’ basic psychological needs satisfaction was assessed using the nine-item BPNS [34]. This instrument posits that the satisfaction with the needs for autonomy, relatedness, and competence is crucial for motivation, optimal development, effective functioning, and good health. BPNS was developed based upon the context of PE [35]. The nine-item BPNS contains three subscales: “perceived competence” (5 items), “autonomy” (2 items), and “relatedness” (2 items). The items were rated on a 7-point Likert scale (1 = strongly disagree to 7 = strongly agree). In this study, the internal consistency reliability was 0.88. 

### 2.3. Analytical Strategy

Structural equation modeling (SEM) was used to test the cross-lagged relationship between the perceived physical literacy and adolescents’ basic psychological needs satisfaction. The authors applied maximum-likelihood estimation. The overall model fit indices were also assessed by the root mean square error of approximation (where RMSEA <0.08 is acceptable), the Tucker–Lewis Index, the comparative fit index (TLI and CFI; values >0.90 are acceptable), and the standardized root mean squared residual (SRMR; <0.08 is acceptable), as suggested by previous research [31,36,37].

In addition, before testing the main hypotheses, we examined the longitudinal measure equivalence of each latent variable [38]. The first step was to build a basic model using confirmatory factor analysis (CFA) in which the error of an item at Time 1 correlated with the corresponding error of the item at Time 2; the authors estimated the correlations among latent variables. The second step was to compare the χ^2^ value of the basic model with the model constrained by factor loadings to be equal across time [32,39,40]. If the χ^2^ difference test was not significant, then the data supported the longitudinal measure equivalence.

To test our hypotheses, the reciprocal model was used to test the bidirectional relationship between PPLI-A and BPNS. In the model, we estimated the cross-lagged effects between PPLI-A and BPNS on each other’s Time 2 counterparts. We also estimated the stability of PPLI-A and BPNS, and the relationship between the Time 1 measurements and the Time 2 follow ups. Specifically, the model consisted of four latent variables, three measures of PPLI-A, and three measures of BPNS and their corresponding items. Corresponding measurement errors across time for each observed variable were correlated. In addition, the residual error of latent variables at the same time was allowed to be correlated, which is called synchronous correlation.

## 3. Results

### 3.1. Descriptive Statistics and Preliminary Analysis

The means, standard deviations, and correlations are presented in Table 1. PPLI-A was related to BPNS (*r* = 0.64, *p* < 0.01) at Time 1. Similarly, the data supported the hypothesis that the relationship of PPLI-A was related to BPNS (*r* = 0.62, *p* < 0.01) at Time 2. Finally, the correlations between the Time 1 and Time 2 latent variables were also in the expected direction (as shown in Table 1).

All the models were estimated by a maximum likelihood estimator using structural equation modeling (SEM). We employed the maximum likelihood statistic for model comparison of invariance analysis and main hypothesis testing. Before fitting the structural models, measurement invariance of the PPLI-A and BPNS across time was first tested respectively as an important precondition for interpreting the longitudinal models [32,37,40,41]. If measurement invariance existed, the participants interpreted the PPLI-A and BPNS in the present study equally across time. For this purpose, we estimated models with the corresponding factor loadings, factor covariances, and residuals of each latent variable, fixed to be equal at the two time points.

Table 2 presents the results of model fits and comparisons. First, the PPLI-A values are (χ^2^ (69) = 497.00, *p* < 0.05; △χ^2^ = 30.34, *p* < 0.05; RMSEA = 0.07; SRMR = 0.055; CFI = 0.93; ΔCFI = 0.007; TLI = 0.93; ΔTLI = 0.026) and the TBPNS values are (χ^2^ (69) = 442.61, *p* < 0.05; △χ^2^ = 43.16, *p* < 0.05; RMSEA = 0.06; SRMR = 0.046; CFI = 0.95; ΔCFI = 0.006; TLI = 0.96; ΔTLI = 0.013). All models have satisfactory values on the fit indices. 

Moreover, we followed the suggestion of Byrne [42] to test the measurement invariance comparisons between models. The results reveal that all the χ*^2^* difference in residuals invariance test (Δχ^2^) were significant, indicating that the invariance was not fully supported. However, research suggests that the invariance test can be evaluated by using other indices; that is, when the value of ΔCFI is equal to or lower than 0.01 [40], and the value of ΔTLI is lower than 0.05 [40]. In the current findings, all the ΔCFI (0.001–0.007) and ΔTLI (0.007–0.026) values were acceptable, indicating satisfactory psychometric properties for measurement invariance, as shown in Table 2. In sum, the preliminary measurement equivalence for both PPLI-A and BPNS was acceptable over time, which means that the techniques measured the constructs in consistent ways at both time points [32,39,40]. Furthermore, we examined the longitudinal measure equivalence of a sample of females and males for each latent variable of the PL and BPNS. The results reveal that for PL, all the ΔCFI (0.003–0.018) and ΔTLI (0.003–0.019) values were acceptable, and that for BPNS all the ΔCFI (0.006–0.018) and ΔTLI (0.003–0.011) values were acceptable [39,40], indicating satisfactory psychometric properties for measurement invariance. These findings indicate that, overall, the model was not significantly different for female and male.

### 3.2. Hypothesis Testing

**Figure 1:**
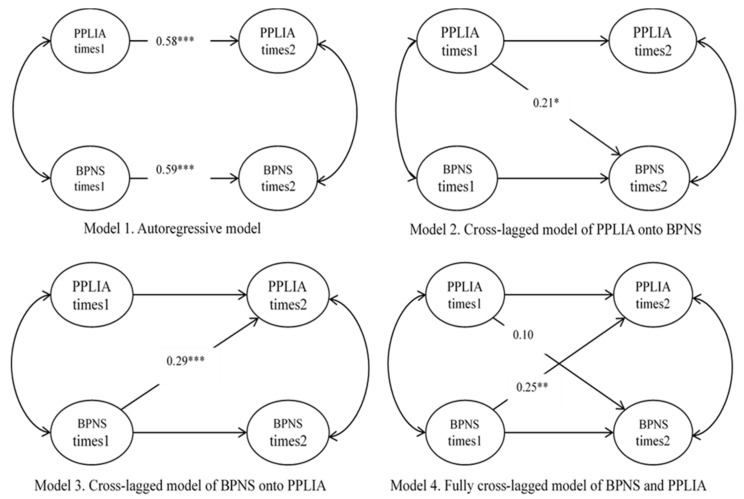
The conceptual path models. * *p* < 0.05; ** *p* < 0.01; *** *p* < 0.001.

To assess the longitudinal associations between PPLI-A and BPNS, we next conducted comparisons between a series of nested cross-lagged models (see Figure 1). The starting point was the autoregressive model (M1), which estimates the stability of the constructs over time [30]. The model (M1) was shown to fit well (χ^2^ (44) = 151.57, *p* < 0.05; RMSEA = 0.06; SRMR = 0.043; CFI = 0.97; TLI = 0.95; ECVI = 0.41). The results of Model 1 indicate that both PPLIA (standardized coefficients *β* = 0.58, *p* < 0.001) and TBPNS (standardized coefficients *β* = 0.59, *p* < 0.001) exhibited significant stability effects from Time 1 to Time 2.

In the second model (M2), the cross-lagged pathway was added from PPLI-A at Time 1 to TBPNS at Time 2. The model (M2) showed appropriate fit to the data (χ^2^ (43) = 146.19, *p* < 0.05; RMSEA = 0.06; SRMR = 0.040; CFI = 0.97; TLI = 0.95; ECVI = 0.40). All parameter estimates in the model (M2) were significant (*p* < 0.05). While controlling the stability effects, the cross-lagged effect of PPLI-A at Time 1 on BPNS at Time 2 was significant (standardized coefficients *β* = 0.21, *p* = 0.018). For the third model (M3), the relationship was reversed, and the path leading from BPNS at Time 1 to PPLI-A at Time 2 was specified. The model (M3) showed appropriate fit (χ^2^ (43) = 140.26, *p* < 0.05; RMSEA = 0.06; SRMR = 0.036; CFI = 0.97; TLI = 0.96; ECVI = 0.39). All parameter estimates in the model (M3) were significant (*p* < 0.001). Specifically, while controlling the stability effects, the M3 path from BPNS at Time 1 to PPLI-A at Time 2 was significant (standardized coefficients *β* = 0.29, *p* < 0.001).

**Figure 2:**
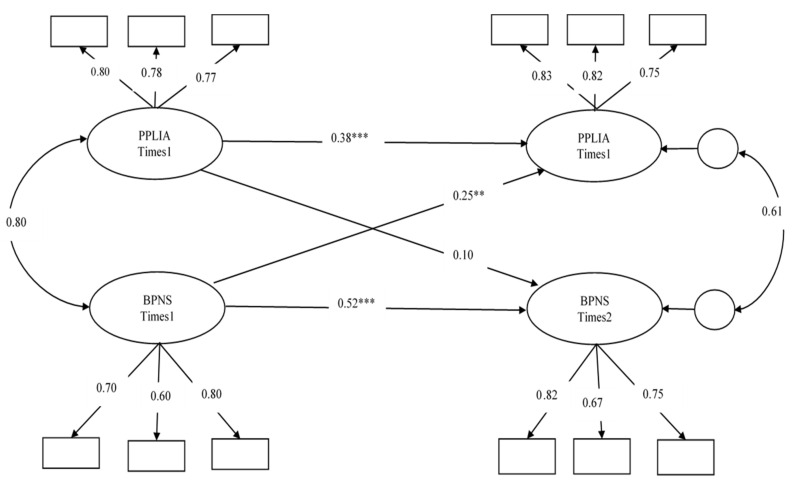
The full cross-lagged conceptual models. This figure only presents paths significant at *p* < 0.05; it omits inter correlations between error variances for simplicity.

Finally, M4 shows the fully cross-lagged model, which included the autoregressive paths linking the same constructs across time points and the cross-lagged paths between PPLI-A and BPNS. The model (M4) was shown to fit well (χ^2^ (42) = 139.09, *p* < 0.05; RMSEA = 0.06; SRMR = 0.035; CFI = 0.97; TLI = 0.95; ECVI = 0.39). In the model (M4), while controlling the stability effect of each latent variable, the path from BPNS at Time 1 to PPLI-A at Time 2 was significant (standardized coefficients *β* = 0.25, *p =* 0.008); however, the path from PPLI-A at Time 1 to BPNS at Time 2 was non-significant (standardized coefficients *β* = 0.10, *p* = 0.278) (see Figure 2).

Although the above results reveal that BPNS at Time 1 was a significant predictor of PPLI-A at Time 2, the inconsistent findings from PPLI-A at Time 1 to BPNS at Time 2 between M2 and M4 need to be further examined. Based on the suggestion of past studies [e.g., 41,42], we thus estimated an additional cross-lagged model that constrained the two cross-lagged paths to be equal and compared this model with Model 4 (the full cross-lagged Model). This result indicates that this constrained model also provided a good fit to the data (χ^2^ (43) =140.65, *p* < 0.05; RMSEA = 0.06; SRMR = 0.036; CFI = 0.97; TLI = 0.96). Moreover, we found a non-significant chi-square difference between this constrained model and the unconstrained model (M4) (△χ^2^ = 1.55, △df = 1, *p* < 0.05), suggesting that the two cross-lagged paths did not significantly differ from each other. According to the model comparisons above, the findings indeed provide overall support for a bidirectional relationship between PPLI-A and BPNS. In other words, we cannot reject the possibility of a bidirectional relation, supporting our expectations (see Table 3).

## 4. Discussion

Through this study, we aimed to investigate the reciprocal relationship mechanism between BPNS and PL using a two-wave longitudinal design. Our findings demonstrate that reciprocal relationships have been identified between BPNS and PL in PE over time. Overall, our hypotheses were supported and provide several contributions to the literature.

The present study expands the BPNS research because we adopted a longitudinal design to observe the changes in students’ BPNS rather than reporting static associations. Furthermore, most of the previous research focused on conceptualizing PL in the PE setting while studying the subject operationally [11,43,44]. It should be noted that the present study clarified the change in the reciprocal relationship between BPNS and PL at two time points. However, our study demonstrated another pathway, which is the reciprocal process. For instance, reciprocity plays a very important role, as the service providers (e.g., PE teachers) need to make sure that the physical activities they are providing stimulate the interest of their participants. The reciprocal process suggested that PL clarifies individuals’ internal motivation which fulfills their BPNS and further promotes their well-being. This result corresponds to the claim that BPNS should be pursued to fulfill intrinsic motivation rather than to achieve extrinsic standards [2,45]. In addition, PL has been characterized by ‘the motivation to capitalize on innate movement potential to make a significant contribution to the quality of life’ (p. 12) [34], which is in line with the SDT, which suggests that these personal dispositions would make a significant contribution to the personal BPNS quality [16,17,18,19,20].

This finding has important theoretical implications because it implies that BPNS may exert an effect on PL. We found that a reciprocal relationship between PL and BPNS exists in a PE domain. Therefore, many studies have regarded PL as a factor influencing athletic abilities, and most studies have confirmed that it is an important concept for assessing students’ athletic abilities. However, based on the SDT perspective, PL is the individual’s ability to perform in a PE-specific domain, and may also affect the student’s BPNS state in the PE domain. These findings parallel the previous conceptions of PL as a holistic unity of the personal conception of movement and understanding of how to respond to movement (e.g., motor skills) [10,27]. By contrast, PL is represented as the environmental, educational, and/or social contexts that could aid in an individual’s BPNS [24]. In sport, PL’s operational conceptualization places importance on the individual’s autonomous application of movement (e.g., understanding of sports and the continuous participation in physical activities, and also involves the development of athletic abilities and the promotion of healthy psychology), conception of movement, and response to adversity [29]. Each different individual BPNS state provides a unique conception of PL as an important disposition acquired by individuals to maintain physical activity throughout the course of one’s life. Motivation serves as an embraced component in both PL and BPNS which provides a powerful source of the psychological needs that all human beings are keen to fulfill in their life [46]. The motivation processes should also be considered in the engagement of physical activities, mostly for intrinsic values such as enjoyment, self-actualization and a sense of competency [34], and further contribute to living a wholesome and flourishing human life [3]. According to Whitehead [3], the constituent traits of human flourishing are autonomy in exercising independence and self-determination for shaping one’s life, and positive self-esteem and acceptance of personal potential. These constituent traits may well fit into the innate needs for autonomy, competence and relatedness, which are essential for positive human growth and optimal functioning in SDT [1,2,47].

## 5. Limitations and Future Directions

While the study has a clear strength over existing research in using a two-time design, some potential limitations must be acknowledged [43]. First, the use of data from only one source increases the risk of common method variance. However, using a two-wave design with an 18-week time interval should reduce the risk concerning common method variance, as respondents are unlikely to remember their prior evaluations over such a time period. Second, the current study did not include an empirical examination of the two key rationales (e.g., explain the influence of students’ different BPNS status on PL), as well as verification of the two conducted via the method of experimental design in the physical activity scenario. Therefore, we suggest that future research should investigate the moderating role of PL variables, such as by using an experimental design to manipulate and investigate their influence on students’ psychological processes and outcomes. Hence, this study first uses the cross-lagged model for design, then to conduct verification to provide powerful evidence for future research. Third, the sample of this study consisted entirely of university students. The results might not be generalized to a different age population, such as primary school or secondary school students. Fourth, the instrument PPLI-A that we used in this study is a self-report measure on a individual’s perception of PL. It has no measurement of movement competence, which is a fundamental aspect of PL. Future research may consider using more objective measures of PL that have been validated against other measures and/or outcomes of PL. Finally, as a longitudinal study, there were only two time points in the current study, so the data representability might be problematic. We recommend that future research addresses this issue by experience sampling while replicating and/or extending our study.

## 6. Conclusions

This study is one of the very few to use cross-lagged panel design to measure two variables at two times and to document the simultaneous occurrence of PE class and its associated participation in physical activity characteristics among students while recognizing the bidirectional effects both PL and BPNS. The study provided several important insights not only for PL and BPNS literature but also for sport educators and learners. In line with this view, we verified that because PL skills are goal-directed skills that help people to shape their emotions, cognitions and behaviors, BPNS should in turn lead to greater self-confidence in terms of helping students to experience a greater sense of self-endorsement of their behavior in school. In addition, they would have enhanced relatedness, belonging, and genuine connection with teachers and peers, and a sense of competence by enabling them to effectively interact with the PE environment and maximize opportunities to express or develop their capabilities and strengths. Since this study used an advanced statistical analysis design to examine the bidirectional effects of BPNS and PL over time in a university PE context, to our knowledge, there have been no similar research studies carried out to date. We therefore could not compare against such studies. However, this study provides evidence-based results to confirm that motivation, as an embraced component of both BPNS and PL, could be widely used in exploring students’ learning outcomes in PE classes, extra-curricular sport activities, and engaging in physical activity within school [16].

In summary, our study contributes to the literature in several ways. First, we expand the understanding of BPNS by investigating whether the context-specific variable of PL is associated with BPNS in PE classes. Second, the possibility that individuals’ PL acquired from PE class can be shaped by BPNS was tested, providing practical insights for educators and learners. Third, we recognize the bidirectional effects of BPNS and PL, which is an important topic in the PE literature [22,23]. With these potential contributions in mind, a longitudinal study with cross-lagged design was conducted to investigate the reciprocal relationship between BPNS and PL in the PE context.

Overall, this study highlights the importance of BPNS for PL in the sports context. The different BPNS status might shape individual PL in PE, and multiple methods are warranted to uncover the role of PL in PE. The present study opens a new avenue in understanding bidirectional effects between BPNS and PL. This is, however, is just a beginning in PE as the study’s conclusions are temporary, and this speculation requires further investigation.

## Figures and Tables

**Table 1:** Descriptive statistics and correlation matrix of variables.

	M	SD	Gender	Age	T1PL	T1TBPNS	T2PL	T2TBPNS
Gender	1.53	0.50	-					
Age	18.35	0.87	−0.06	-				
T1PL	3.77	0.65	−0.15 *	−0.03	-			
T1BPNS	3.81	0.66	−0.22 *	−0.02	0.64 **	-		
T2PL	3.83	0.66	−0.18 *	0.01	0.56 **	0.45 **	-	
T2BPNS	3.86	0.67	−0.15 *	0.02	0.43 **	0.56 **	0.62 **	-

Note. *n* = 594. * *p* < 0.05; ** *p* < 0.01. T = Time; PL = physical literacy; BPNS = The basic psychological needs satisfaction. Entries in italics on the diagonal are the Cronbach’s alpha reliability coefficients for the PPLIA scales and the bivariate correlations of the two calling items respectively.

**Table 2:** Model fit of various invariance models.

Model	χ^2^	DF	Δχ^2^	ΔDF	SRMR	RMSEA	CFI	ΔCFI	TLI	ΔTLI
PPLIA										
Configural	453.83 *	48	-	-	0.05	0.08	0.93	-	0.90	-
Measurement weights	461.03 *	54	7.21	6	0.05	0.08	0.93	0.001	0.91	0.011
Stuctural covariances	466.67 *	60	5.64	6	0.05	0.07	0.93	0.002	0.92	0.020
Measurement residuals	497.00 *	69	30.34 *	9	0.05	0.07	0.93	0.007	0.92	0.026
BPNS										
Configural	385.90 *	48	-	-	0.04	0.07	0.96	-	0.94	-
Measurement weights	387.51 *	54	1.61	6	0.04	0.07	0.96	0.001	0.95	0.007
Stuctural covariances	399.45 *	60	11.93	6	0.04	0.06	0.96	0.001	0.95	0.011
Measurement residuals	442.61 *	69	43.16 *	9	0.04	0.06	0.95	0.006	0.95	0.013

Note. *n* = 594. PPLIA = Perceived physical literacy instrument for adolescents; BPNS = The basic psychological needs satisfaction; * *p* < 0.05.

**Table 3:** Cross-lagged standardized regression paths and autoregressive paths.

Model	χ^2^	*df*	χ^2^/*df*	Δχ^2^	Δ*df*	TLI	CFI	SRMR	RMSEA	ECVI
						>0.90	>0.95	<0.08	<0.06	
Default model (M1)	151.57 *	44	3.45	12.48 *	2	0.95	0.97	0.04	0.06	0.41
CLPPLIA (M2)	146.19 *	43	3.40	7.10 *	1	0.95	0.97	0.04	0.06	0.40
CLBPNS (M3)	140.26 *	43	3.26	1.64	1	0.96	0.97	0.03	0.06	0.39
FULLCL (M4)	139.09 *	42	3.31	-	-	0.95	0.97	0.03	0.06	0.39

Note. *n* = 594. Default model = only autoregression effects. CLPPLIA = only cross-lagged effect of physical literacy–the basic psychological needs satisfaction congruence on physical literacy with auto regression effects and correlations between factors at the same time point; CLBPNS = only cross-lagged effect of the basic psychological needs satisfaction on physical literacy–physical literacy congruence (the reverse to model 2) with auto regression effects and correlations between factors at the same time point; FULLCL = full cross-lagged model with all bidirectional effects and synchronous correlations between factors at the same time point; TLI = Tucker-Lewis index; CFI = comparative fit index; SRMR = standardized root mean square residual; RMSEA = root mean square error of approximation; ECVI = Expected cross-validation index. In relation to χ2, models 1, 2, and 3 are compared to model 4; * *p* < 0.05.
